# BHLHE40 Regulates IL-10 and IFN-*γ* Production in T Cells but Does Not Interfere With Human Type 1 Regulatory T Cell Differentiation

**DOI:** 10.3389/fimmu.2021.683680

**Published:** 2021-07-07

**Authors:** Molly Javier Uyeda, Robert A. Freeborn, Brandon Cieniewicz, Rosa Romano, Ping (Pauline) Chen, Jeffrey Mao-Hwa Liu, Benjamin Thomas, Esmond Lee, Alma-Martina Cepika, Rosa Bacchetta, Maria Grazia Roncarolo

**Affiliations:** ^1^ Department of Pediatrics, Division of Stem Cell Biology and Regenerative Medicine, Stanford School of Medicine, Stanford, CA, United States; ^2^ Stanford Institute for Stem Cell Biology and Regenerative Medicine, Stanford School of Medicine, Stanford, CA, United States; ^3^ Center for Definitive and Curative Medicine, Stanford School of Medicine, Stanford, CA, United States

**Keywords:** T cell, regulatory, cytokine, transcription factor, transcriptome

## Abstract

Type 1 regulatory T (Tr1) cells are subset of peripherally induced antigen-specific regulatory T cells. IL-10 signaling has been shown to be indispensable for polarization and function of Tr1 cells. However, the transcriptional machinery underlying human Tr1 cell differentiation and function is not yet elucidated. To this end, we performed RNA sequencing on *ex vivo* human CD49b^+^LAG3^+^ Tr1 cells. We identified the transcription factor, BHLHE40, to be highly expressed in Tr1 cells. Even though Tr1 cells characteristically produce high levels of IL-10, we found that BHLHE40 represses IL-10 and increases IFN-γ secretion in naïve CD4^+^ T cells. Through CRISPR/Cas9-mediated knockout, we determined that *IL10* significantly increased in the sgBHLHE40-edited cells and BHLHE40 is dispensable for naïve CD4^+^ T cells to differentiate into Tr1 cells *in vitro*. Interestingly, BHLHE40 overexpression induces the surface expression of CD49b and LAG3, co-expressed surface molecules attributed to Tr1 cells, but promotes IFN-γ production. Our findings uncover a novel mechanism whereby BHLHE40 acts as a regulator of IL-10 and IFN-γ in human CD4^+^ T cells.

## Introduction

Regulatory T (Treg) cells are multifaceted immunomodulatory cells that are composed of two subgroups: thymus- and peripherally- derived (1). Type 1 regulatory T (Tr1) cells are a peripherally-derived subset that is induced under tolerogenic conditions in an antigen-specific manner (2). Tr1 cells regulate the function of other T cells directly and indirectly through antigen presenting cells predominately by secreting high levels of the pleiotropic cytokine IL-10 (3–5). Unlike other IL-10 producing T helper cells, such as Th2 and Th17 cells, Tr1 cells have a unique cytokine profile: high IL-10 and TGF-β, low IL-2, no IL-4 and IL-17, and variable amounts of IFN-γ (2). Besides IL-10 production, murine and human Tr1 cells can be identified by the co-expression of CD49b and LAG3 (5). Sole expression of either LAG3 or CD49b is insufficient to identify Tr1 cells because these markers are expressed on other cells such as activated effector T cells (6) and Th17 cells (7), respectively. CD49b^+^LAG3^+^ Tr1 cells are highly suppressive *in vitro* and *in vivo* (5). They represent approximately 1-5% of the memory CD4^+^ T cells in the peripheral blood of humans (8). This low Tr1 cell frequency in the periphery has posed challenges for studying them as compared to their FOXP3^+^ Treg counterparts, which are more abundant and constitutively express high levels of FOXP3, a master transcription factor (9).

While a master transcription factor has not been identified in Tr1 cells, the importance of many transcription factors has been demonstrated in Tr1-cell biology. Specifically, mouse models (10, 11) and IL-27 dependent Tr1 cell differentiation (12) studies have identified transcription factors such as EGR2 (13, 14), BLIMP1 (15), AHR (5, 16–18), EOMES (19), IRF1/BATF (20), and IRF4 (21–23). Despite model specific caveats, the dominant role of IL-10 in Tr1 cell differentiation and function suggests that the molecular machinery controlling Tr1 cell identity is IL-10-centric. The prevailing theory of IL-10 regulation in mouse CD4^+^ T cell subsets is that Basic Helix-Loop-Helix Family Member E40 (BHLHE40) inhibits *IL10* while c-Maf activates *IL10* (24). While a genomic repressor and enhancer has been identified near the *IL10* transcriptional start site for BHLHE40 (25) and c-MAF (26) respectively, their expression is inversely correlated in c-MAF and BHLHE40 single-knockout models (27), suggesting both direct and indirect IL-10 mediated regulation. In mouse models, BHLHE40 regulates the immune response to resolve *Toxoplasma gondii* (28) and *Mycobacterium tuberculosis* infections by downregulating IL-10 and upregulating IFN-γ (25). BHLHE40 additionally has a broader regulatory role over cytokine expression in other T cell subsets and disease contexts. In response to *Heligmosomoides polygyrus*, it ensures expression of Th2 cytokines such as IL-4 and IL-5 (22) and during graft-versus-host disease, it mediates gastrointestinal inflammation *via* GM-CSF production (29). The multifunctional role of BHLHE40 in different CD4^+^ T cell subsets, including Tr1 cells, has yet to be delineated in human cells.

Here we show the first full transcriptomic characterization of *ex vivo* isolated human Tr1 cells, which identified that human Tr1 cells differentially express high levels of *BHLHE40*. We characterized BHLHE40’s role in primary human CD4^+^ T cells using CRISPR-Cas9 to generate BHLHE40-deficient cells, a Tr1 cell induction protocol to differentiate allogenic (allo) antigen-specific Tr1 cells *in vitro*, and a temporally controlled gene system to overexpress BHLHE40. We showed that BHLHE40 can regulate the co-expression of Tr1 cell surface molecules, CD49b and LAG3, but it is not required for *in vitro* differentiation of allo-antigen specific Tr1 cells from naïve CD4^+^ T cells. BHLHE40 was also required for efficient secretion of IFN-γ, IL-4, and IL-2 and robust proliferation of naïve and memory CD4^+^ T cells. Despite upregulation of the canonical Tr1 cell surface markers by BHLHE40 overexpression, the upregulation of IL-10 was triggered only after knocking out BHLHE40 in naïve CD4^+^ T cells. Overall, these data demonstrate that BHLHE40 regulates phenotypic Tr1 cell markers and two key cytokines, IFN-γ and IL-10, underscoring the interconnected network of BHLHE40 in Tr1 cell and underlying CD4^+^ T cell biology.

## Materials And Methods

### Primary Human Cells

All human peripheral blood mononuclear cells (PBMC) were obtained from buffy coats of de-identified healthy donors (Stanford Blood Center, Palo Alto, CA, USA) and all cord blood samples were obtained from the negative fraction of mononuclear cells stained with CD34 microbeads placed through a LS column (Miltenyi Biotec) (Stanford Binns Program for Cord Blood Research, Palo Alto, CA, USA). All material was collected in accordance with IRB guidelines.

### RNA Sequencing

20,000 Tr1 cells (LiveDead Aqua-/CD3^+^/CD4^+^/CD45RA^-^/LAG3^+^/CD49b^+^) and non-Tr1 T memory cells (LiveDead Aqua^-^/CD3^+^/CD4^+^/CD45RA^-^/LAG3^-^/CD49b^-^) were isolated by sorting *via* FACS directly into two independent tubes per cell type from each donor containing Trizol-LS. Nucleic acid was isolated from the aqueous phase after centrifuging with chloroform and RNA was further isolated through concentrating using linear polyacrylamide solution (Sigma Aldrich) and digesting with RQ1 RNAse-free DNAse (Promega). RNA was then cleaned with RNeasy MinElute Cleanup Kit (Qiagen). RNA integrity was checked using the RNA 6000 Pico kit on a bioanalyzer (Agilent). cDNA prep was performed with Nugen Ovation RNAseq system V2. cDNA was sheared using a sonicator (Covaris). Libraries were generated with NEBNext Ultra DNA for Illumina kit. Libraries were normalized and pooled before paired-end 151bp sequenced on Illumina NextSeq500.

### RNA Sequencing Processing and Analysis

RNA sequencing (RNA-seq) transcripts were trimmed using Skewer (v0.2.2), dual passed aligned using STAR (v2.5.3a) to GRCh38. Tabulated gene counts were imported into DESeq2 (v1.16) to perform exploratory analysis, visualization, and differential gene expression using R. The datasets presented in this study can be found online in NCBI Gene Expression Omnibus under the accession number: GSE169761.

### Lentivirus Production

BHLHE40 cDNA ORF was purchased from Sino Biological and cloned into pCW57.1 using cut sites NheI and AgeI. pCW57.1 was a gift from David Root (Addgene plasmid # 41393; http://n2t.net/addgene:41393; RRID : Addgene_41393). 293T cells were transfected with pMD2.G, pMDLg/pRRE, pILVV01, and pAdvantage (Promega, Madison, WI, USA) using TransIT^®^-LT1 (Mirus Bio, USA) and concentrated by ultracentrifugation as previously described (30, 31). Titer was estimated by limiting dilution on 293T cells by puromycin resistance after transduction of 3 days followed by 2 days of puromycin selection.

### Cell Isolation and Culturing

Naïve CD4^+^ T cells were isolated from CD34^-^ umbilical cord blood cells collected by the Binns Program for Cord Blood Research or from healthy adult peripheral blood by using the EasySep™ Human Naïve CD4^+^ T Cell Isolation Kit (STEMCELL Technologies, USA); >95% CD3^+^/CD4^+^/CD45RA^+^ cells were obtained. CD4^+^ T cells were isolated from PBMC using EasySep Human CD4^+^ T Cell Isolation Kit (STEMCELL Technologies, USA) and >95% CD3^+^/CD4^+^ T cells were obtained. CD4^+^ T cells were maintained using a base T cell media of 5% Human AB serum (Milipore-Sigma) in X-VIVO-15 (Lonza) and 50U/mL IL-2 which was refreshed every 3-4 days in addition to any otherwise noted supplements.

### Differentiating Allo-Antigen Specific Tr1 Cells

CD14^+^ cells were isolated from PBMCs using the manufacturer’s protocol for CD14^+^ microbeads (Miltenyi) and differentiated into mature DC (matDC) or DC-10 as previously described (32) with the following minor changes: CD14^+^ cells were cultured in a base media using 10% human AB serum and matDC were differentiated with 5µg/mL of synthetic monophosphoryl lipid A (InvivoGen) on day 5. Cells were collected on day 7 and irradiated at 6,000 rads. Naïve CD4^+^ T cells were isolated from a donor allogenic to the CD14^+^ cell donor used and then co-cultured at a 10:1 ratio (T cell:DC) for 10 days. The control allo-antigen specific T cells cultured with matDC (“T-allo cells”) were cultured without cytokine supplements and the T cells culture with DC-10 that ultimately become the *in vitro* induced allogenic-antigen specific Tr1 cells (“T-allo10 cells”) were cultured with 10ng/mL rhIL-10 added on day 0 and day 5 (32, 33). Cells were collected after 10 days of co-culture.

### Overexpression of BHLHE40 in Naïve CD4^+^ T Cells

1x10^6^ CD3^+^CD4^+^CD45RA^+^ T cells were stimulated as previous described (30) with the addition of 10ng/mL rhIL-10 (BD Biosciences, USA) and transduced with lentivirus in the presence of 4µg/mL polybrene and 1µg/mL doxycycline (Mirus Bio, USA). 24 hours later, the media was replaced with 50U/mL IL-2. 10ng/mL rhIL-10 was added every 4 days for the first 12 days, 1µg/mL doxycycline was added every two days. Transduced cells were selected for puromycin resistance after culturing with puromycin 1µg/mL for 4 days. Cells were expanded using an irradiated allogenic feeder mixture (1:10 ratio of JY cell line to human PBMC with 1µg/mL soluble CD3).

### Knockout of BHLHE40

When using frozen CD4^+^ T cells, cells were thawed in T cell media overnight with 50U/mL IL-2 before resuspending in P3 solution (Lonza) while freshly isolated CD4^+^ T cells were directly resuspended in P3 solution at 10^6^ cells/20µL and nucleofected with 54.9 pmol of Hi-Fi Cas9 (Integrated DNA Technologies) and 33 pmol of each sgRNA (Synthego) using the 4D-16 well strips using program EO-115 (Lonza). No differences were observed in editing efficiency or downstream characterization between fresh and frozen starting T cells (data not shown). Cells were plated directly into T cell media containing 50U/mL IL-2 after nucleofection. After 2 days, cells were stimulated using 10µL/1mL of ImmunoCult Human CD3/CD28/CD2 (STEMCELL Technologies). After 3 days, media was replaced with T cell media by removing the majority of existing media containing the soluble stimulant. 12 days after activation, cells were collected and further characterized.

### Insertion and Deletion (Indel) Analysis

gDNA was isolated with Quick Extract (Lucigen) following the manufacturer’s protocol. PCR primers were designed approximately 500bp surrounding the sgRNA cut sites and used to amplify gDNA for Sanger sequencing. Indels were calculated by comparing edited and mock edited cells using Inference of CRISPR Edits [Synthego’s Performance Analysis, ICE Analysis. 2019. V2.0. Synthego; (January 2020)].

### Western Immunoassay (Wes)

1x10^6^ T cells were collected 14 days after stimulation and whole-cell extracts were prepared in RIPA lysis buffer containing a protease inhibitor cocktail (Roche, complete Mini Protease Inhibitor Cocktail). Protein concentrations were determined with the BCA protein assay reagent (Pierce). Samples were run on the Wes (ProteinSimple) according to the manufacturer’s protocol. 0.5µg of protein lysate was loaded per lane and the following antibodies were used: DEC1 (Novus Biologicals, NB1001800SS, 1:50) and HSP90 (Cell Signaling Technology, 4877, 1:1000). Image analysis was conducted using the Compass for SW software.

### Cytokine

To measure cytokine secretion upon stimulation, 1x10^5^ T cells were incubated for 24-48h in 96-well round-bottomed plates pre-coated with immobilized anti-CD3 (10 μg/mL) and soluble anti-CD28 (1 μg/mL). The levels of secreted IL-2, IL-4, IL-10, and IFN-γ were determined by ELISA with technical duplicates (BD Biosciences). To measure intracellular cytokine production, 1x10^5^ T cells were incubated for 5 hours in 96-well round-bottomed plates containing Leukocyte Activation Cocktail, with GolgiPlug (BD Biosciences) or 10ng/mL Brefeldin A (Biolegend) only. Cells were then fixed, permeabilized, stained using Fixation/Permeabilization Solution Kit (BD Biosciences), and read on a flow cytometer.

### Proliferation

Cells were labeled with 5µM Carboxyfluorescein succinimidyl ester (CFSE) for 5 mins at room temperature then washed once with fetal bovine serum and once with PBS before resuspending at 10^6^ cells/mL in T cell media. 50,000 cells were plated in a 96-well round-bottomed plate with CD3/CD28 Dynabeads (ThermoFisher Scientific) at 1 bead per 20 cells and dye dilution was measured on a flow cytometer on the indicated days.

### Anergy

T-allo and T-allo10 cells were labeled with CFSE as described in the Proliferation methods section and plated in a 96-well round-bottom plate with the following conditions: alone with 50,000 cells per well, irradiated matDC at a 10:1 ratio (T-allo/10: matDC) with 55,000 total cells per well, and 50,000 cells with 2,500 CD3/CD28 Dynabeads (ThermoFisher Scientific). 3 days later, cells were stained for CD3, CD4, and DAPI, and measured on a flow cytometer to quantify the CFSE dye dilution in the CD4^+^ live T cell gate. CFSE proliferation gates were set using the T-allo/10 CFSE of the cells plated alone. Anergy was calculated with the following equation: (% proliferated T-allo mock - % proliferated T-allo10 mock or sgBHLHE40)/% proliferated T-allo mock.

### qPCR

T cells were washed twice in PBS and then resuspended in 300µL of RLT buffer containing beta-mercaptoethanol. Rneasy Plus Micro Kit (Qiagen) was used according to the manufacture’s guidelines to further isolate RNA. RNA was quantified using Qubit RNA HS Assay Kit (ThermoFisher Scientific) or a NanoDrop Spectrophotometer (ThermoFisher Scientific). cDNA was generated using SuperScript IV VILO Master Mix (ThermoFisher Scientific). *BHLHE40*, *IFN-γ, IL-4, IL-10, IL-2*, and housekeeping genes including: *RPLPO*, *GAPDH*, and β*-Actin* were measured using TaqMan Gene Expression Assays with TaqMan Gene Expression Master Mix (ThermoFisher Scientific) in a 384 well plate with an Applied Biosystems qPCR machine. Relative fold gene expression was calculated using the 2^–ΔΔ^ **^Ct^ **method.

### Flow Cytometry

Antibodies used in these experiments can be seen in **Supplementary Table 1**. Fluorescence activated cell sorting was performed using a BD FACSAria Special Order Research Product. Other flow cytometry assays that did not require sorting were performed on a Cytoflex (Beckman Coulter) or BD FACSAria. Flow cytometry data analysis was performed using FlowJo v10 (FlowJo, Version 10, Ashland, OR, USA).

### Statistical Analysis

For the non-RNA-seq-derived data, statistical analysis was performed using GraphPad Prism 7. The center bars and whiskers indicate the mean with standard deviation. The data were analyzed using a non-parametric test that do not assume equal variances between groups such as the Wilcoxon test for groups of 2 paired samples unless otherwise noted. Multiple testing correction was applied when applicable.

## Results

### Tr1 Cells Express High Levels of *BHLHE40 In Vivo*

**Figure 1:**
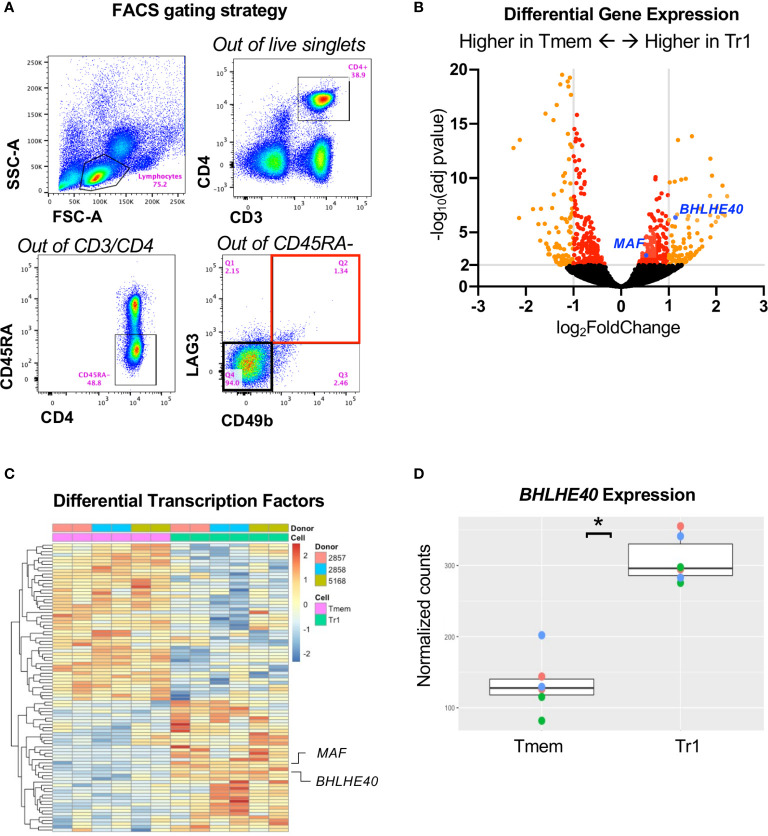
RNA sequencing identifies BHLHE40 in Tr1 cells. **(A)** Sorting strategy for *ex vivo* human Tr1 cells. Healthy donor PBMCs were gated for CD3^+^/CD4^+^/CD45RA^-^. Tr1 cells were additionally gated and sorted for LAG3^+^/CD49b^+^, indicated with a red bounding box, while non-Tr1 Tmem cells were sorted for LAG3^-^/CD49b^-^, indicated with a black bounding box. **(B)** Volcano plot of differentially expressed genes between Tr1 and non-Tr1 Tmem cells. Dots indicate individual genes, with genes having an adjusted p value < 0.05 indicated in red and with genes with also an abs(log2(fold change)) > 1 in orange. *MAF (*cMAF) and *BHLHE40* are indicated with blue dots. **(C)** Differentially expressed transcription factors between Tr1 and non-Tr1 Tmem cells with an adjusted p value < 0.05 are plotted by rows and CD4^+^ T cell samples by columns. Fill color is z-scaled per gene. **(D)** Normalized counts (variance stabilized normalized counts) of BHLHE40 gene expression between Tr1 and non-Tr1 Tmem cells. *adjusted p value < 0.05 after Benjamini–Hochberg multiple testing correction. n = 3 healthy donors, biological duplicates for Tr1 and Tmem cells. Tmem, non-Tr1 T memory.

To understand the transcriptome of *ex vivo* human Tr1 cells in an unbiased, high-throughput approach, we RNA-sequenced sorted Tr1 cells with the CD3^+^CD4^+^CD45RA^-^CD49b^+^LAG3^+^ phenotype (**Figure 1A**; Q2 outlined in red), and the non-Tr1 memory T cells (Tmem; CD3^+^CD4^+^CD45RA^-^CD49b^-^LAG3^-^; **Figure 1A**; Q4 outlined in black) directly from the peripheral blood of healthy donors without any *in vitro* activation. We identified 1,058 genes that were significantly differentially expressed between Tr1 and Tmem cells, with an adjusted p value < 0.05 and out of those, 139 genes were overexpressed > 2-fold in Tr1 cells (**Figure 1B**). In our effort to identify a master transcription factor in Tr1 cells, analogous to FOXP3 in FOXP3^+^ Tregs, we identified 90 differentially expressed transcription factors with an adjusted p value < 0.05 (**Figure 1C** and **Supplementary Table 2**). From this differentially expressed transcription factor gene list, we confirmed higher expression of cMAF, the IL-10 activator encoded by *MAF* (17) in Tr1 cells (**Figure 1C**). To our surprise, *BHLHE40*, an IL-10 repressor (28, 34), was also overexpressed by Tr1 cells (**Figure 1B**; blue dot) compared to non-Tr1 Tmem cells (**Figure 1D**).

### BHLHE40 Overexpression Promotes a Partial Tr1 Cell Phenotype

**Figure 2:**
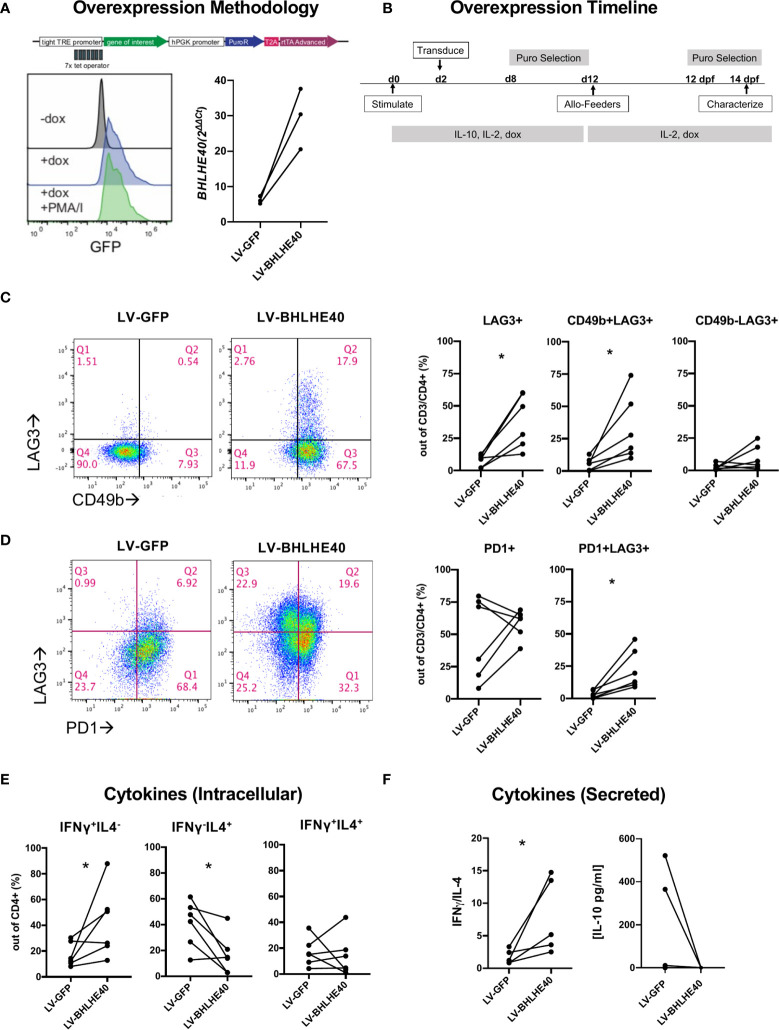
Overexpression of BHLHE40 induces co-expression of CD49b and LAG3 in naïve CD4^+^ T cells. **(A)** Validation of a dox inducible system for transgene overexpression in CD4^+^ T cells. pCW57.1 construct used for expressing GFP or BHLHE40 (top). GFP expression in CD4^+^ T cells is induced after addition of dox. Transgene expression is maintained during activation. Dox was incubated for 4 hours and cells were immediately collected and checked for GFP expression by flow cytometry (Bottom-left). BHLHE40 overexpression confirmed by qPCR. GFP and BHLHE40 transduced CD4^+^ T cells were incubated with dox for 4 hours and RNA was collected. *BHLHE40* RNA was measured using qPCR Taqman probes along with housekeeping genes*: RPLPO, GAPDH*. n = 3 (Bottom-right). **(B)** Timeline of dox-inducible BHLHE40 in naïve CD4^+^ T cells. CD4^+^ naïve T cells were cultured with IL-10 and then expanded on allo-feeders with IL-2 for 14 days before being characterized. **(C, D)** Surface phenotype of transduced cells after 2 rounds of allo-feeder expansion. Gated on live, CD3^+^, CD4^+^ cells **(C)** CD49b and LAG3 expression. Representative plots of CD49b and LAG3 in one donor (Left). Summarized data of CD49b and LAG3 expression on BHLHE40 overexpressing cells compared to GFP expressing cells. (Right). n = 6. **(D)** Co-expression of LAG3 and PD1. Representative plots of PD1 and LAG3 in one donor (Left). Summarized data of PD1 and LAG3 expression on BHLHE40 overexpressing cells compared to GFP expressing cells (Right). n = 6. **(E, F)** Increased Th1 phenotypic cytokines in BHLHE40 overexpressing cells. **(E)** Increased intracellular IFN-γ^+^/IL4^-^ cells in BHLHE40 overexpressing cells. Intracellular cytokine staining of IFN-γ and IL-4 in BHLHE40 overexpressing cells after 5 hours of PMA/I and Brefeldin A n = 6. Ratio paired t-test. **(F)** Increased secretion of IFN-γ. Cells are stimulated for 48 hours with plate-bound αCD3 and soluble αCD28.Cytokines in the supernatant were quantified by ELISA. IFNγ/IL-4 ratios (Left). Absolute IL-10 concentrations (Right). n = 5, Ratio paired t test. PMA/I, phorbol-myristate-acetate/ionomycin, Allo-feeders = allogenic-PBMC feeders; Dox, doxycycline; dpf, days post feeder; puro, puromycin. *p value < 0.05.

To understand the function of high BHLHE40 expression in Tr1 cells, we generated a BHLHE40 overexpression model. We utilized a doxycycline (dox) inducible lentiviral system to enable temporal control of BLHLE40 and GFP expression (**Figure 2A**; top). We transduced naïve CD4^+^ T cells with lentivirus containing the *BHLHE40* transgene (“LV-BHLHE40”) alongside a control lentivirus containing *GFP* (“LV-GFP”). We validated transgene expression in CD4^+^ T cells after 4 hours of dox exposure and confirmed that expression was constant after T cell activation (**Figure 2A**; bottom-left). Even though BHLHE40 is endogenously expressed, we confirmed dox-inducible expression of *BHLHE40* at the RNA level by collecting RNA for qPCR analysis after 4 hours of dox exposure in transduced cells (**Figure 2A**; bottom-right). After validating the overexpression system, we designed a workflow for generating and expanding a pure population of dox-responsive cells (**Figure 2B**). We next investigated if BHLHE40 overexpressing cells and GFP control cells differentially expressed surface markers consistent with a Tr1 cell phenotype (**Figure 2C**; left panel, **Supplementary Figure 1**). We found that overexpressing BHLHE40 led to a significant increase in LAG3^+^ cells compared to GFP controls, and this was restricted to the CD49b^+^ population (**Figure 2C**; right panel). To test if any other co-inhibitory molecules were modulated by BHLHE40 overexpression, we co-stained for PD1 (**Figure 2D**; left). While overall PD1 expression was not affected, PD1^+^LAG3^+^ co-expression significantly increased after BHLHE40 overexpression (**Figure 2D**; right). To see if BHLHE40 overexpressing cells adopted a Tr1 cell cytokine profile, we checked intracellular cytokine expression and cytokine secretion after stimulation. We observed a significant increase in intracellular Th1 cytokines (IFN-γ^+^/IL-4^-^), a significant decrease in Th2 cytokines (IFN-γ^-^/IL-4^+^), and no change in the percentage of IFN-γ^+^/IL-4^+^ cells in BHLHE40 overexpressing cells compared to GFP control cells (**Figure 2E**). We saw a similar trend in cytokine secretion whereby the IFN-γ/IL-4 ratio was significantly increased in BHLHE40 overexpressing cells (**Figure 2F**). Interestingly, no detectable IL-10 production was observed in the BHLHE40 overexpressing cells. Even though there is significant induction of co-expressed surface molecules attributed to Tr1 cells, the cytokine profile more closely resembles Th1 cells. Therefore, overexpression of BHLHE40 alone is not sufficient to induce functional Tr1 cells.

### BHLHE40 Is Not Required for Differentiation of Allo-Antigen Specific Tr1 Cells

**Figure 3:**
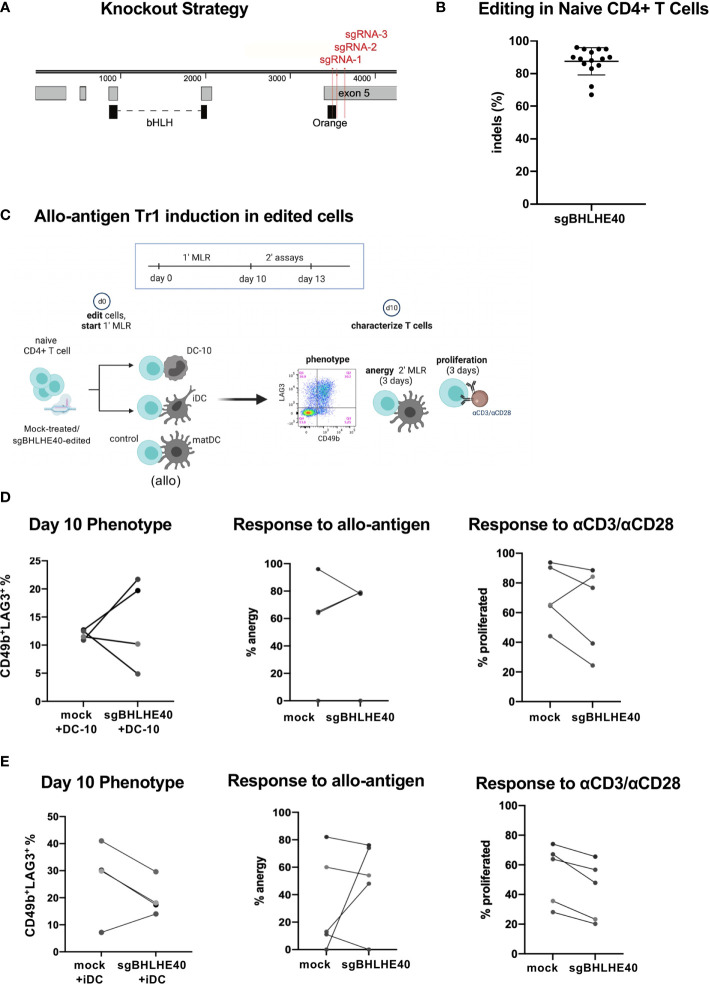
BHLHE40 is not required for generating allo-antigen specific Tr1 cells. **(A)** BHLHE40 knockout strategy. 3 sgRNA (red) were designed to simultaneous target the N’ terminus exon 5 of *BHLHE40.* Protein domains (black) and exons (grey) are annotated. **(B)** High knockout efficiency in naïve CD4^+^ T cells. Indels % were calculated 2-3 days after nucleofection. N = 15 healthy donors. **(C)** Schematic of allo-antigen Tr1 cell induction of sgBHLHE40-edited cells with iDC, DC-10, or control matDC. **(D, E)** CD4^+^ T cell characterization after primary MLR with **(D)** DC-10 or **(E)** iDC. %Tr1 cells 10 days after primary MLR. Tr1% is measured by flow cytometry and gated on live/CD3^+^/CD4^+^/CD45RA^-^/CD49b^+^/LAG3^+^ cells (left). Allo-antigen specific anergy assay of cells restimulated in a secondary MLR with matDC. % anergy of mock-treated and sgBHLHE40-edited (middle). Proliferation response, as indicated by CFSE dye dilution, of mock-treated and sgBHLHE40-edited cells taken at day 10 of the primary MLR after restimulation with Dynabeads for 3 days (right). n = 5 healthy donors.

Next, we wanted to explore the contribution of BHLHE40 during Tr1 cell differentiation. Specifically, we assessed if BHLHE40 was required to generate Tr1 cells *in vitro*. We started by generating BHLHE40-deficient naïve CD4^+^CD45RA^+^ T cells from healthy donors by nucleofecting multiplexed ribonucleoprotein complexes comprised of three single guide RNAs (sgRNA) and Cas9 protein. These 3 sgRNAs targeting *BHLHE40* (sgBHLHE40) (**Figure 3A**) were predicted to have minimal off-target effects and high activity (35) (data not shown). The multiplexed strategy was designed to generate large deletions in exon 5 which contains the orange motif of the BHLH-orange domain that is critical for DNA binding (36–38) (**Figure 3A**). We disrupted the *BHLHE40* locus efficiently with an average 87.5% (± 8.07% SD) insertion and deletion rate as determined by Sanger sequencing and ICE analyses (**Figure 3B**). Notably, ICE analyses revealed that the *BHLHE40* locus harbored a 36-base pair (bp) or a 144 bp genomic deletion (**Supplementary Figure 2A**; top) caused by high combinatorial cutting activity of sgRNA-2/sgRNA-3 and sgRNA-1/sgRNA-3 respectively (**Supplementary Figure 2A**; bottom blue arrows). We also confirmed on the protein level that there was a 90% (± 6.7% SD) reduction in total wild-type protein. Importantly, the wild type BHLHE40 band could also contain the 36bp deleted-mutant BHLHE40 that leads to only a minor 1.4kDa shift which cannot be visually resolved. Our protein analyses also demonstrated evidence of truncated protein, as expected (**Supplementary Figure 2B**; green box). Notably, the donor with the least efficient gene disruption (65% genomic knockout) still exhibited a 78% protein reduction (**Supplementary Figure 2C**).

Having established a robust knockout strategy, we combined our BHLHE40 knockout system with the protocol for differentiating allo-antigen specific Tr1 cells (32, 33) in order to specifically investigate the contribution of BHLHE40 in the development of Tr1 cells (**Figure 3C**). We co-cultured sgBHLHE40-edited naïve CD4^+^ T cells in a primary mixed lymphocyte reaction (MLR) with allo-tolerogenic dendritic cells called DC-10 (32, 33). As a negative control for Tr1 cell induction, we used allo-mature myeloid dendritic cells (matDC) in the primary MLR. At the end of the Tr1 cell induction protocol, we saw an increase in CD49b^+^LAG3^+^ Tr1 cells as measured by the surface phenotype (33), with no differences in the percentages between the sgBHLHE40-edited and the mock-treated cells (**Figure 3D**; left). To assess if there was a functional difference in the acquisition of allo-antigen specific anergy, we restimulated the cells at the end of the 10-day induction in a secondary MLR with matDC derived from the same donor as the DC-10 cells. We saw that after the secondary MLR, both mock-treated cells and sgBHLHE40-edited T cells had weak proliferation, indicating no change in the acquisition of allo-antigen specific anergy (**Figure 3D**; middle). To confirm that both the sgBHLHE40-edited cells and the mock-treated cells had the capacity to proliferate, we stimulated them with αCD3/αCD28. Interestingly there was a decreasing trend in proliferation response to αCD3/αCD28 stimulation in the sgBHLHE40-edited cells (**Figure 3D**; right). These results were validated in a similar *in vitro* model, using allo-immature myeloid dendritic cells (iDC), which express low levels of IL-10 (32, 33). All of the findings we observed with the DC-10 cells were recapitulated when using iDC in the primary MLR, including the induction of CD49b^+^LAG3^+^ Tr1 cells, allo-antigen specific anergy, and proliferative response to αCD3/αCD28 stimulation (**Figure 3E**). Combined, these data suggest that BHLHE40 is not required for *in vitro* induction of CD49^+^LAG3^+^ Tr1 cells that mediate allo-antigen specific anergy.

### BHLHE40 Deficiency Increases IL-10, Decreases IFN-γ and IL-2 Production, and Reduces Proliferation in Naïve CD4^+^ T Cells

**Figure 4:**
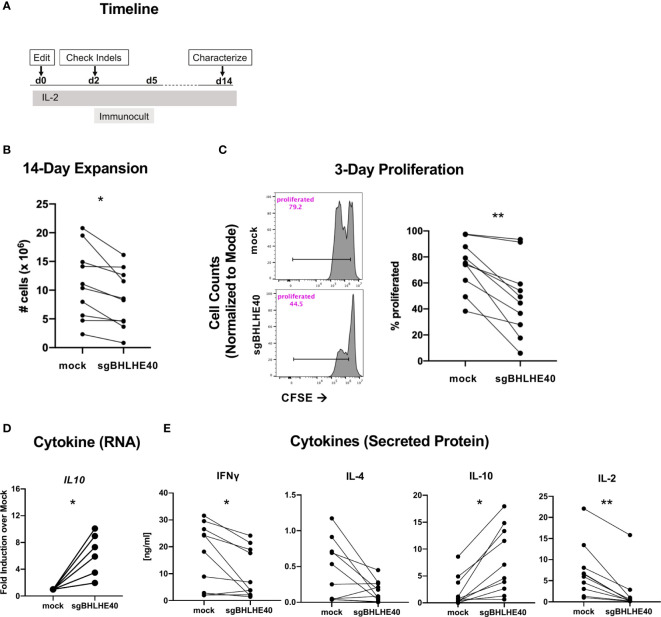
BHLHE40 deficiency increases IL-10 in naïve CD4^+^ T cells. **(A)** Timeline of generating BHLHE40-edited CD4^+^ T cells. **(B)** *In vitro* expansion of mock-treated and sgBHLHE40-edited naïve CD4^+^ T cells. Cells were collected and counted with an automated cell counter after 2, 14-day expansions with Immunocult. n = 15. **(C)** Representative flow cytometry histogram of CFSE dilution 3 days post αCD3/αCD28 Dynabead stimulation. Gated on live singlets (left). Cumulative CFSE proliferation data of all samples (right). **(D)** Cytokine gene expression. *IL10* expression 6 hours after stimulation with αCD3/αCD28 Dynabeads. Expression is normalized to the house keeping gene, *RPLPO*. n = 6. **(E)** T cells were stimulated with plate-bound αCD3 and soluble αCD28, and supernatant was collected at 24h (IL-2) and 48h (IFN-γ, IL-4, IL-10) to measure cytokines by ELISA. n = 10, *p value < 0.05, **p value < 0.01, Wilcoxon matched-pairs signed rank test.

To better probe the pathways controlled by BHLHE40 in T cells more broadly, we next characterized the growth and cytokine production of sgBHLHE40-edited naïve CD4^+^ T cells using the same BHLHE40 knockout strategy as above (**Figure 3A**). After expanding the cells by polyclonal stimulation (**Figure 4A**), we observed a significant reduction of cell recovery (**Figure 4B**) and a significant decrease in cell divisions, measured by CFSE dilution, in the sgBHLHE40-edited cells (**Figure 4C**). These findings were consistent with the reduced proliferation we observed after restimulating allo-antigen specific sgBHLHE40-edited T cells with αCD3/αCD28, but with greater magnitude. To see if disrupting BHLHE40 induced a cytokine profile opposite from when we overexpressed BHLHE40, we checked cytokine expression after αCD3/αCD28 stimulation and found that *IL10* significantly increased in the sgBHLHE40-edited cells (**Figure 4D**). However, IFN-γ, IL-2, and IL-4 were unchanged (**Supplementary Figure 3**). When we further investigated cytokine production in sgBHLHE40-edited cells, we specifically measured cytokine secretion after αCD3/αCD28 stimulation. We observed a significant decrease in IFN-γ and IL-2 in the sgBHLHE40-edited cells (**Figure 4E**). While IL-4 levels were lower in the sgBHLHE40-edited cells, the reduction was not significant, most likely due to the mock-treated cells having an overall low baseline of IL-4 secretion (**Figure 4E**). Importantly, the sgBHLHE40-edited cells had a significant increase in IL-10 secretion, which was consistent with gene expression data (**Figure 4E**). When unstimulated, both sgBHLHE40-edited and mock-treated cells exhibited no IL-10 secretion in the detectable range (data not shown). The decrease in secreted IL-2 is aligned with the observed reduction in proliferation of the sgBHLHE40-edited cells. These findings are consistent with previous observations in *CD4*-conditional *Bhlhe40* knockout mice (28) and highlight a conserved function of BHLHE40 in naïve human and mouse CD4^+^ T cells.

### BHLHE40 Is Required for Optimal IFN-γ and IL-4 in CD4^+^ T Cells

**Figure 5:**
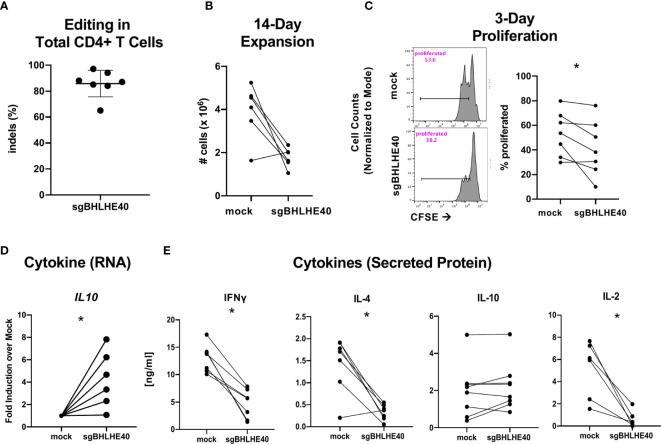
BHLHE40 knockout leads to a reduction in IFN-γ and IL-4 expression. **(A)** High knockout efficiency in total CD4^+^ T cells. **(B)** *In vitro* expansion. After 2, 14-day stimulations with Immunocult, cells were collected and counted with an automated cell counter. **(C)** Representative flow cytometry histogram of CFSE dilution 3 days post αCD3/αCD28 Dynabead stimulation. Gated on live singlets (left). Cumulative CFSE proliferation data of all samples (right). **(D)** Cytokine gene expression. *IL10* expression 6 hours after stimulation with αCD3/αCD28 Dynabeads. Expression is normalized to the house keeping gene, *RPLPO*. n = 6. **(E)** Cytokine Protein Secretion. T cells were stimulated with plate-bound αCD3 and soluble αCD28 and supernatant was collected at 24h (IL-2) and 48h (IFN-γ, IL-4, IL-10) to measure cytokines by ELISA. n = 7, *p value < 0.05, Wilcoxon matched-pairs signed rank test.

While naïve CD4^+^ T cells are known to rapidly upregulate BHLHE40 after stimulation (39), we also observed that non-Tr1 Tmem cells expressed notable amounts of *BHLHE40*, albeit at lower levels, than Tr1 cells (**Figure 1D**). Accordingly, we sought to determine the effect of BHLHE40 on total human CD4^+^ T cells. We utilized the same BHLHE40 knockout and expansion strategy in total CD4^+^ T cells as in naïve CD4^+^ T cells (**Figure 4A**) and achieved similar knockout rates (**Figure 5A**). We observed a significant reduction in long-term cell expansion *in vitro* (**Figure 5B**) and a decreased induction of cell division after αCD3/αCD28 stimulation (**Figure 5C**). Similar to the gene expression phenotype observed in sgBHLHE40-edited naïve CD4^+^ T cells, *IL10* expression after αCD3/αCD28 stimulation significantly increased in the sgBHLHE40-edited cells compared to mock-treated cells (**Figure 5D**), while *IFN-γ, IL-2*, and *IL-4* were unchanged (**Supplementary Figure 4**). On the protein level, sgBHLHE40-edited cells secreted less IL-2, IFN-γ, and IL-4 (**Figure 5E**). In stark contrast to our findings in naïve CD4^+^ T cells and *IL10* mRNA expression, we did not detect a significant difference in IL-10 secretion in our sgBHLHE40-edited total CD4^+^ T cell model (**Figure 4E**). The consistent reduction in proliferative capacity after BHLHE40 knockout despite no change in IL-10 secretion suggests that anti-proliferative effects of BHLHE40 deficiency are more likely caused by a reduction in IL-2 secretion and less likely due to IL-10 secretion. Ultimately, our data show that BHLHE40 is required for optimal secretion of IFN-γ, IL-4, and IL-2 in addition to proliferation. While our observations showcase the conserved function of BHLHE40 in regulating IFN-γ and IL-4, the inverse relationship between IFN-γ and IL-10 expression at the protein or RNA level was not recapitulated in the sgBHLHE40-edited cells in total CD4^+^ T cells (25, 28).

## Discussion

Our collective results delineate the role of the transcription factor, BHLHE40, using *in vitro*-induced models of human Tr1 cells and CD4^+^ T cells. While we began by unbiasedly investigating the transcriptome in primary human CD49b^+^LAG3^+^ Tr1 cells (5) and non-Tr1 Tmem CD4^+^ T cells from peripheral blood using high throughput RNA sequencing, we ultimately focused on BHLHE40, an *IL-10* inhibitor (28, 34), because it was highly expressed in Tr1 cells together with an *IL-10* activator cMAF (17, 27). Interestingly, circulating Tr1 cells expressed higher levels of BHLHE40 compared to the non-Tr1 Tmem cells. We investigated this contrasting pattern of expressing both an IL-10 activator and inhibitor by genetically manipulating BHLHE40 using knockout and overexpression systems. We found that BHLHE40 modulates the production of cytokines IFN-γ, IL-4, IL-2, and IL-10, promotes proliferation, and induces the surface expression of CD49b and LAG3 in primary human naïve CD4^+^ T cells. However, BHLHE40 is dispensable for the differentiation of naïve CD4^+^ T cells into allo-antigen specific Tr1 cells *in vitro.*

Past efforts to characterize the transcriptome of human Tr1 cells utilized microarrays of stimulated and unstimulated human Tr1-cell clones (5) and bulk RNA-sequencing of stimulated human IL-10 producing CD4^+^ T cells (40). In public datasets, *BHLHE40* is expressed highly in both non-IL-10 producing cells and IL-10 producing cells, but not in FOXP3^+^ Tregs (40), suggesting that BHLHE40 is a transcriptional regulator that is utilized by many memory CD4^+^ T cell subsets, excluding FOXP3^+^ Tregs. While past methodology of utilizing mouse models to investigate transcription factors during immune development has been fruitful, there are intrinsic limitations and biological differences that must be delineated (41). Ultimately by utilizing CRISPR-Cas9 technologies and lentiviral overexpression vectors in primary human T cells, we have been able to resolve species-specific discrepancies and identify new transcriptional control mechanisms surrounding human Tr1 cell biology.

BHLHE40 tightly regulates multiple cytokines in human T cells, which we showed through knockout and overexpression models. We observed that knocking out BHLHE40 reduced IFN-γ production, and overexpressing BHLHE40 promoted IFN-γ production, which suggests BHLHE40 is required in human CD4^+^ T cells for efficient production of IFN-γ, consistent with what is reported in mouse (25, 28). Interestingly, IL-4 remained downregulated regardless of if BHLHE40 was knocked out or overexpressed, which could be due to BHLHE40 indirectly controlling IL-4 expression through intermediate regulators not explicitly captured in this study. IL-10 production only increased when we knocked out BHLHE40 in naïve and not in total CD4^+^ T cells. However, *IL10* mRNA was upregulated in both naïve and total CD4^+^ T cells after knocking out BHLHE40, which suggests that in the absence of BHLHE40, a post-transcriptional mechanism downregulating IL-10 exists in total CD4^+^ T cells that is not active in naïve CD4^+^ T cells. In addition, IL-2 was consistently downregulated in both BHLHE40 knockout naïve and total CD4^+^ T cells. Chromatin immunoprecipitation of BHLHE40 in mouse (25) and Jurkat cells (42) suggests that BHLHE40 has minimal to no binding near *IL-2*. Therefore, we predict that BHLHE40 more likely represses IL-2 production through intermediate regulators because many of the gene regulators in the *IL-2* promoter are well conserved (43). Interestingly, the reduction in IL-2 was observed alongside a proliferation defect in BHLHE40 knockout cells. A similar proliferation defect was previously described in stimulated naïve CD4^+^ T cells from *Bhlhe40* germline knockout mice (44) and shRNA-based BHLHE40 knock down in Jurkat cells (39). However, in naïve CD4^+^ T cells taken from CD4-conditional *Bhlhe40* knockout mice, cell recovery after αCD3/αCD28 stimulation and cell proliferation by CFSE was unchanged (28). One explanation for the dissimilarities in these results is that BHLHE40 is expressed at other developmental time points and depending on the mouse genetic model, there are molecular changes that affect the ability of BHLHE40 knockout cells to proliferate. For instance, it was previously reported that Cre expression can affect CD4^+^ T cell development (45). The advantage of our system is that we disrupt BHLHE40 in cells from healthy donors after the T cell compartment is established, so we do not risk introducing unintentional developmental defects.

Despite being expressed by memory CD4^+^ T cells, the high expression of BHLHE40 in Tr1 cells makes it a multifunctional target whose expression could be modulated for therapeutic purposes. Even though Tr1 cells express multiple co-inhibitory molecules that are typically associated as being T cell exhaustion markers, such as LAG3 and PD1, they are persistent cells that are fully functional when taken out of the body (5, 46). RNA sequencing of murine CD4^+^ T cells previously revealed that BHLHE40 is highly expressed in IL-10^+^ cells with a co-inhibitory phenotype, classified as CD49b, LAG3, and IL-10^eGFP^ triple-positive cells (46). In corroboration of these findings, we demonstrated that while BHLHE40 was not required for Tr1 cell differentiation, its overexpression did promote upregulation of the coinhibitory receptor, LAG3, in addition to CD49b. This increase in LAG3 and CD49b co-expression is most likely due to some indirect gene regulation as ChIP-seq data of overexpressed *BHLHE40* in Jurkat cells did not lead to any enriched genomic binding (42). Moreover, we showed that upon overexpressing BHLHE40 in naïve CD4^+^ T cells, there is an increase in the IFN-γ to IL-4 ratio but no IL-10 production. In this context, BHLHE40 enforces efficient expression of cytokines such as IFN-γ, which could provide a protective role against T cell exhaustion, such as what has been described for CD8^+^ tumor infiltrating T cells (47). The involvement of BHLHE40 in different T cell subsets opens up questions surrounding BHLHE40 modulation of Tr1 cells *in vivo.* Currently, studies on the *in vivo* functions of BHLHE40 have largely focused on the pro-inflammatory contributions of non-Tr1 T cells in the gastrointestinal tract (28, 29) and the nervous system (39). Therefore, tracking the explicit expression patterns of BHLHE40 in Tr1 cells at steady-state and during disease to see if BHLHE40 modulation contributes to disease manifestation would be invaluable.

Following differentiation, Tr1 cells express high levels of IL-10 and consequently upregulate BHLHE40, which we demonstrate leads to increased CD49b and LAG3 co-expression and IFN-γ production, recapitulating the phenotype and cytokine production profile of *in vitro*-derived and circulating Tr1 cells. While we showed that BHLHE40 suppressed IL-10 production by naïve CD4^+^ T cells, it was unable to do so in total CD4^+^ T cells despite modulating IL-10 transcription in these cells. Given that Tr1 cells exist within the memory compartment, it is likely they possess a mechanism to overcome the BHLHE40-mediated suppression of IL-10. Accordingly, we posit that BHLHE40 acts in concert with other mechanisms to control Tr1 cell phenotype and function. Of note, it remains to be determined whether BHLHE40 overexpression persists in CD49^+^LAG3^+^ Tr1 cells after homing to specific tissues where they exert the suppressive function. Alternatively, the unique polyfunctionality of circulating Tr1 cells, which produce both IL-10 and IFN-γ, may be essential to suppress undesirable immune responses and for host protection against severe inflammatory responses.

## Data Availability Statement

The datasets presented in this study can be found in online repositories. The names of the repository/repositories and accession number(s) can be found below: Gene Expression Omnibus (GEO), accession code GSE169761.

## Author Contributions

Contributions: MU, RF, A-MC, RB, RR, and MR designed the research. MU, RF, BC, PC, JM-HL, BT, RR, and EL performed experiments. MU, RF, and MR analyzed the data. MU, RF, and MR wrote the paper. All authors contributed to the article and approved the submitted version.

## Funding

MU was supported by an NSF DGE (1147470) and Blavatnik Family Fellowship. BC was supported by the Maternal and Child Health Research Institute Postdoctoral Fellowship. RF was supported by NIH T32 (T32HL007952). JM-HL was supported by NIH T32 (2T32DK098132-06A1) and the Maternal and Child Health Research Institute Postdoctoral Fellowship.

## Conflict of Interest

The authors declare that the research was conducted in the absence of any commercial or financial relationships that could be construed as a potential conflict of interest.
