# Supplementary material for: BHLHE40 Regulates IL-10 and IFN-γ Production in T Cells but Does Not Interfere With Human Type 1 Regulatory T Cell Differentiation
Source: Front Immunol. 2021 Jul 7;12:683680. doi: 10.3389/fimmu.2021.683680 (PMC8293608; doi:10.3389/fimmu.2021.683680)
Supplement: Supplementary file 2 [file DataSheet_2.pdf]

Supplementary Material

Supplementary Table

Supplemental Table 1. Antibodies.xlsx

Supplemental Table 2. Differentially Expressed Transcription Factors.csv

Supplementary Figures

Supplementary Figure 1

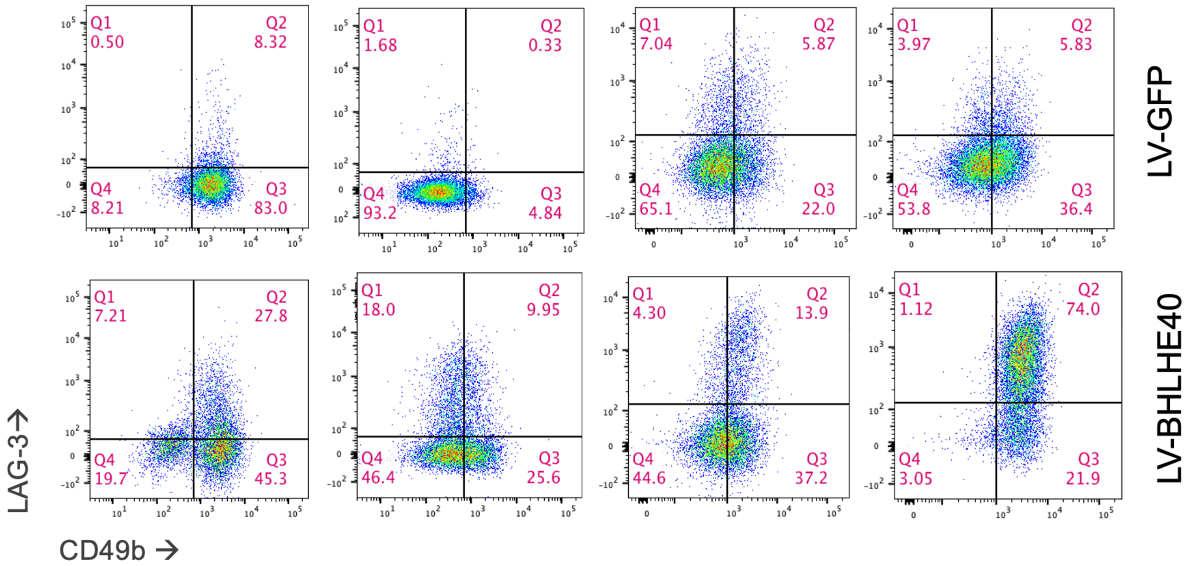

Supplementary Figure 1. CD4$^+$ naïve T cells were cultured with IL-10 and then expanded on allo-feeders with IL-2 for 14 days before being characterized. Surface phenotype of transduced cells after 2 rounds of allo-feeder expansion. Gated on live, CD3$^+$, CD4$^+$ cells. CD49b and LAG3 expression. Independent donors are shown in columns. The first two columns and the last two columns are donors from two independent overexpression experiments with flow cytometry data acquired on separate days.

Supplementary Figure 2

A **Characterization of Knockout (genomic)**

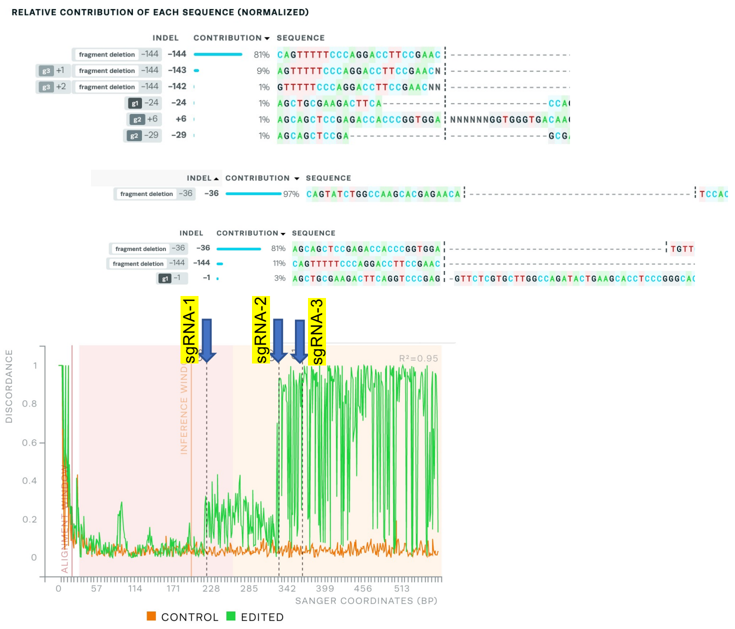

B **Characterization of Knockout (protein)**

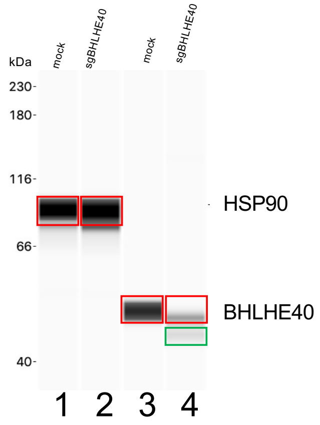

C **Knockout Summary**

DonorID	T cell origin	protein KO	genomic KO
82	total	78%	65%
100	total	89%	88%
292	naïve	83%	89%
3207	naïve	93%	85%
3204	naïve	94%	83%
2169	naïve	97%	94%
3203	naïve	97%	86%

Supplementary Figure 2. Validation of BHLHE40 knockout strategy. (A) (Top) Representative examples of genomic editing results using 3 multiplex guides against *BHLHE40* using the tool: Inference of CRISPR Edits (ICE). (Bottom) ICE discordance data of sgBHLHE40-edited samples in green and mock-treated samples in orange. (B) (Left) Protein Simple Wes generated images of separated protein lysates. Lanes 1 and 2 were run with HSP90 antibody and lanes 3 and 4 were run with BHLHE40 polyclonal antibody with the following parameters: 0.5µg protein/lane, separation time 55 mins, exposure time 5s. (Right) Summarize data of genomic and protein knockouts (KO). Protein knockout was calculated by dividing the normalized area under the curve (BHLHE40/HSP90) for sgBHLHE40-edited samples by mock-treated samples. n= 7

Supplementary Figure 3

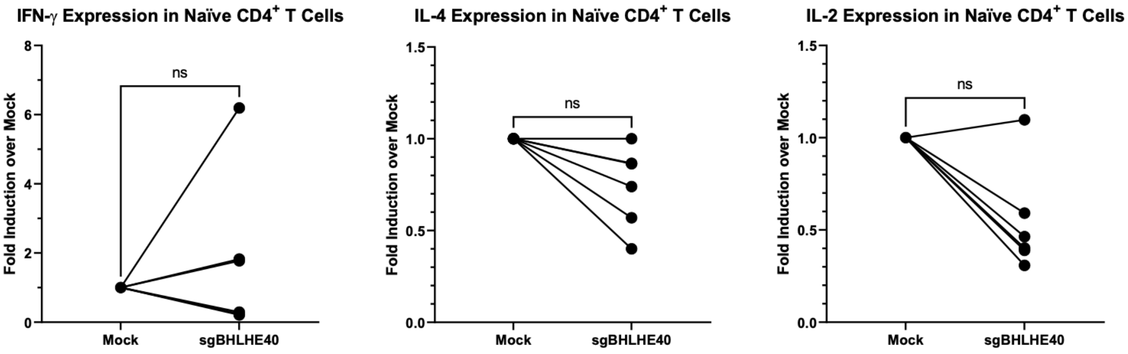

Supplementary Figure 3. Cytokine gene expression in naïve CD4+ T cells. Following 2 rounds of expansion with allogeneic feeder cells, mock-treated or sgBHLHE40-edited naïve CD4+ T cells were stimulated with aCD3/aCD28 Dynabeads. RNA was collected 6 hours post-stimulation and was reverse transcribed. IFN-γ, IL-4, and IL-2 were quantified in the resulting cDNA using qPCR Taqman probes and normalized to the housekeeping gene, RPLPO. n = 6. ns = not significant. Wilcoxon matched-pairs signed rank test.

Supplementary Figure 4

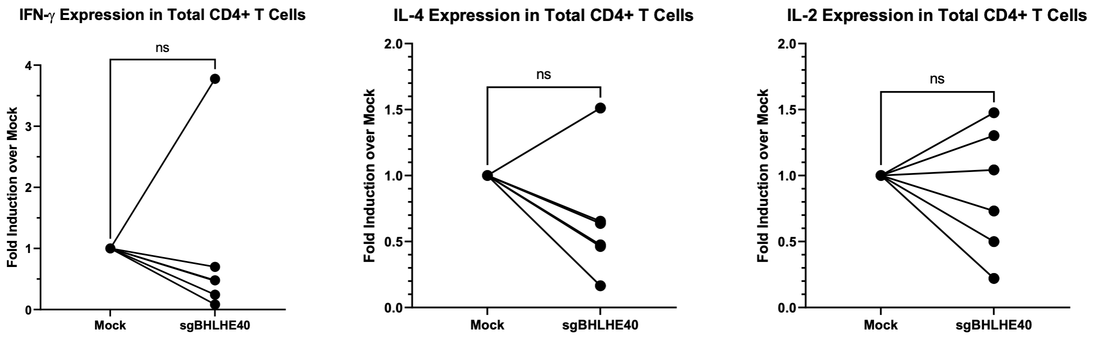

Supplementary Figure 4. Cytokine gene expression in total CD4+ T cells. Following 2 rounds of expansion with allogeneic feeder cells, mock-treated or sgBHLHE40-edited total CD4+ T cells were stimulated with aCD3/aCD28 Dynabeads. RNA was collected 6 hours post-stimulation and was reverse transcribed. IFN-γ, IL-4, and IL-2 were quantified in the resulting cDNA using qPCR Taqman probes and normalized to the housekeeping gene, RPLPO. n = 6. ns = not significant. Wilcoxon matched-pairs signed rank test.
